# Clinical significance of elevated serum soluble CD40 ligand levels as a diagnostic and prognostic tumor marker for pancreatic ductal adenocarcinoma

**DOI:** 10.1186/1479-5876-12-102

**Published:** 2014-04-21

**Authors:** Hye Won Chung, Jong-Baeck Lim

**Affiliations:** 1 Department of Internal Medicine, Division of Gastroenterology, International ST. Mary’s Hospital, Incheon, Republic of Korea; 2 Department of Laboratory Medicine, Yonsei University College of Medicine, Seoul, Republic of Korea

**Keywords:** Biomarker, Diagnostic, Pancreatic cancer, Prognostic, Soluble CD40 ligand

## Abstract

**Background:**

CD40-CD40 ligand (CD40L) interaction is considered to contribute to the promotion of prothrombotic responses and production of angiogenesis-associated factor in addition to adaptive immune responses. Recently, the role of soluble CD40L (sCD40L) has gained interest in cancer, although its exact functions remain unknown. This study evaluated the clinical significance of sCD40L in patients with pancreatic ductal adenocarcinoma (PDAC) and validated its utility as a PDAC diagnostic and prognostic biomarker.

**Methods:**

Serum sCD40L levels were measured by chemiluminescent immunoassay and compared among normal, chronic pancreatitis (CP, high-risk), and PDAC group in both training (n = 25 per group) and independent validation (n = 30, 30, and 55, respectively) datasets through one-way ANOVA test with the post-hoc Bonferroni method. To evaluate the diagnostic potential of serum sCD40L for PDAC, receiver operating characteristic (ROC) curves were generated and logistic regression analysis was conducted. To investigate the sCD40L-assoicated cytokines/chemokines in PDAC, cytokines/chemokines levels were analyzed by a MILLIPLEX MAP Human Cytokine/Chemokine Kit. To assess the prognostic potentials of sCD40L, Kaplan-Meier survival curve and Cox proportional-hazards regression analysis were applied.

**Results:**

Serum sCD40L levels were significantly higher in PDAC group compared with non-cancer groups in both training (*p* < 0.05) and validation (*p* < 0.05) datasets. Clinically, serum sCD40L closely correlated with unresectability (γ_s_ = 0.342, *p =* 0.011) and distant metastasis (γ_s_ = 0.294, *p =* 0.030) of PDAC. ROC curve and logistic regression analysis demonstrated the remarkable predictive potentials of serum sCD40L for PDAC (80.0% sensitivity and 85.5% specificity at cut-off point, 0.45; logistic regression), superior to those of CA19-9 and CEA. According to cytokines/chemokines assay, serum sCD40L levels were closely correlated with serum levels of pro-angiogenic cytokines (EGF, VEGF, IL-8) and immunosuppressive cytokines (IL-6, IL-10, IL-1RA). Kaplan-Meier survival analysis demonstrated patients with high-serum sCD40L (> 35,000 ng/ml) had a poorer prognosis than those with low-serum sCD40L (log-rank, *p* = 0.015). Multivariate Cox regression analysis yielded a hazard ratio of 2.509 (95% CI, 1.038–6.067, *p* = 0.041) for mortality in the high-serum sCD40L group.

**Conclusions:**

Serum sCD40L is correlated with immunosuppression and angiogenesis in PDAC carcinogenesis/progression, and is a promising diagnostic and prognostic biomarker for PDAC superior to CA19-9 and CEA.

## Background

CD40 ligand (CD40L) was initially identified as a T-cell receptor for ligation of the co-stimulatory molecule CD40, and CD40-CD40L interaction was considered as a key event for effective adaptive immune response [1,2]. Subsequently, CD40L was identified on the surface of non-immune cells including activated platelets, endothelial cells, and epithelial cells [3-5]. Activated T-lymphocytes and platelets can release soluble CD40L (sCD40L) into circulation [3], and both membrane-bound CD40L and sCD40L can interact with CD40 which is expressed on vascular cells and activated platelets, resulting in promoting inflammatory and prothrombotic responses [3-6]. Additional studies reported that CD40-CD40L interaction produces many angiogenesis-associated factors, such as vascular endothelial growth factor (VEGF) [7-9]. Like this, early studies mainly focused on the pathophysiological role of CD40L in cardiovascular and immuno-inflammatory diseases [9,10]. However, subsequent other studies found that CD40-CD40L interaction affected tumor cell migration, and then the role of soluble CD40L (sCD40L) has gained interest in cancer [11]. Generally, tumor pro-coagulant activities cause thrombin generation, which induces platelet activation [12,13] and resultant release of angiogenesis-associated cytokines [14]. In cancer patients, sCD40L is more likely derived from activated platelets than from T-cells [15]; therefore, sCD40L can affect cancer development and progression by inducing thrombotic reactions and releasing angiogenesis-associated cytokines. This idea is supported by evidence that cancer patients, including those with pancreatic ductal adenocarcinoma (PDAC), have significant platelet activation [16-18]. Widespread expression of CD40 in human carcinomas also supports the role of this ligand in cancer pathogenesis [19]. Actually, several recent human studies have showed the elevation of serum sCD40L levels in patients with certain solid tumors and myeloproliferative neoplasms [6,20-22]. But its exact role remains elusive yet.

PDAC is one of the most difficult malignancies to diagnose and treat. More than 80% of PDAC are surgically unresectable at diagnosis because of the characteristics of this disease (e.g., rapid progression and proximity to major vessels, etc.) and delayed diagnosis, which can be caused by complex, expensive, and often invasive current conventional diagnostic methods and non-specific vague symptoms of PDAC [23]. Thus, the discovery of useful serum biomarkers for PDAC is clinically valuable.

Although no information is currently available on the clinical significance of sCD40L in PDAC, previous study reporting that CD40 activation in islets and ductal cells due to oxidative stress/inflammation produces various cytokines/chemokines [24] suggests the potential role of sCD40L in PDAC pathogenesis. Further, past studies support the implication of sCD40L in PDAC pathogenesis by exhibiting the cancer-related coagulopathy (Trousseau’s syndrome) in PDAC [18], and by showing the effect of anti-platelet therapy to control PDAC metastasis in nude mice [25].

In current study, we first evaluate the clinical significance of sCD40L in PDAC patients compared with normal healthy controls and patients with chronic pancreatitis (CP, high-risk), and validate serum sCD40L as a potential biomarker for PDAC compared with pre-existing PDAC biomarkers, carbohydrate antigen 19–9 (CA19-9) and carcinoembryonic antigen (CEA) using prospectively collected human serum samples.

## Methods

### Study subjects and sample collection

This study enrolled 190 subjects from the Yonsei University Health System: 75 subjects for initial biomarker identification (training dataset) and following 115 subjects for independent validation. The study datasets included 3 groups according to the PDAC carcinogenic sequence: normal, CP (high-risk), and PDAC.

For the initial training dataset, the sample size was calculated to be 25 subjects per group using Russ Lenth’s interactive power/sample size online calculator because of the lack of information on the mean values for each group. This sample size achieved a statistical power > 80%, assuming that there were 3 comparison groups, the estimated standard deviation (SD) was 1, and the confidence level was 0.05 (one-way analysis of variance [ANOVA]). The independent validation dataset included 30 normal, 30 CP, and 55 PDAC patients. This sample size allowed achievement of statistical power > 90% using the Number Cruncher Statistical System Power Analysis and Sample Size (NCSS PASS) program with the results for mean and SD taken from the training dataset (one-way ANOVA).

As normal controls, we enrolled age- and gender-matched healthy individuals who underwent a medical checkup, and were revealed to have normal pancreas on imaging studies, and had no PDAC risk factors. CP group included patients with typical radiological and/or histopathological findings of CP. All PDAC patients were diagnosed histopathologically using biopsy or surgical specimens. Patients with acute or chronic illnesses such as cardiovascular and immuno-inflammatory diseases were excluded. Those with other cancers and/or other pancreatic malignancies besides ductal adenocarcinoma were also excluded.

TNM classification of PDAC was determined according to the American Joint Committee on Cancer (AJCC) Staging Manual, 6^th^ edition. A pathologist at the Yonsei University Health System reviewed all histopathological information. Blood samples were collected before treatment initiation. Blood samples were stored at -80°C as serum fractions until analysis. The Institutional Review Board of the Yonsei University Health System approved this research, and all participants provided written informed consent.

### Measurement of serum cytokine levels using a chemiluminescent immunoassay

Using a commercially available MILLIPLEX MAP Human Cytokine/Chemokine Kit (Millipore, Billerica, MA, USA), we measured the serum sCD40L levels. We also measured the serum levels of angiogenesis-associated cytokines including epidermal growth factor (EGF), VEGF, and interleukin (IL)-8 and anti-inflammatory immunosuppressive cytokines including IL-6, IL-10, and IL-1 receptor antagonist (IL-1RA). This kit allowed simultaneous quantification of all tested cytokines. Briefly, the filter plate was pre-wetted with 200 μL assay buffer for 10 min at room temperature (RT), followed by vacuum removal of the assay buffer. Twenty-five microliters of standard or control was added to the appropriate well, and 25 μL assay buffer was added to the sample wells, but not the background well. Next, 25 μL of the appropriate matrix solution was added to the background, standard, and control wells, followed by addition of 25 μL sample to appropriate wells. After mixing, 25 μL beads were added, and the plate was incubated overnight at 4°C with shaking. After incubation, the fluid was removed and the plate was washed twice. Detection antibodies (25 μL) were added, and the plate was incubated for 1 h at RT with shaking. Streptavidin-phycoerythrin (25 μL) was added to each well containing 25 μL detection antibodies and was incubated for 1 h at RT with shaking. The fluid was then removed, the plate was washed, and 150 μL sheath fluid was added. After re-suspension for 5 min, the median fluorescent intensity was read on a Luminex 100™ IS and analyzed using the logistic curve-fitting method to determine cytokine concentrations.

### Measurement of serum levels of CA19-9 and CEA

Serum CA 19–9 and CEA levels were measured using quality-controlled Vitros-3600 automatic analyzer (Ortho Clinical Diagnostic, New York, USA) and Beckman Access CEA assay (Beckman Coulter Inc. Chaska, USA), respectively.

### Statistical analysis

All assays were performed in duplicate in a blinded fashion on the same day. Each value is expressed as the mean with 25–75% standard deviation (SD). We used an independent validation dataset to determine the reproducibility of the diagnostic potential of serum sCD40L for PDAC that was determined using the training dataset. One-way ANOVA test with multiple comparisons using the post-hoc Bonferroni method was applied to compare mean serum levels of values among three groups. Receiver operator characteristic (ROC) curves were generated and the area under the curve (AUC) was calculated to compare the diagnostic accuracy of tested markers for predicting PDAC. To validate the diagnostic potential of serum sCD40L, CA19-9, CEA, and combinations of these 3 markers, we performed logistic regression analysis. Each marker was included as a linear term. For comparisons among each panel, the cut-off point ensured a target sensitivity of around 80%. Spearman’s correlation (coefficient, γ_s_) and Pearson’s correlation (coefficient, γ_p_) analysis were performed to assess the correlations between serum values and non-continuous and continuous variables, respectively.

Overall survival was estimated using the Kaplan-Meier method, and the log-rank test was used to compare patients with low-serum sCD40L levels (≤35,000 ng/mL) and those with high-serum sCD40L levels (>35,000 ng/mL) in the validation dataset. Univariate and multivariate Cox proportional-hazards regression models were used to evaluate the prognostic potential of serum sCD40L in the validation dataset.

A gastroenterologist and an MD of laboratory medicine analyzed the data, with support from a statistician who specializes in biomarker studies. *P*-values less than 0.05 (*p* < 0.05) were considered statistically significant. Statistical analyses were performed using IBM SPSS Statistics 20.0 (SPSS Inc, Chicago).

## Results

### Serum sCD40L levels along the PDAC carcinogenic sequence in training set: comparison with serum CA19-9 and CEA

Serum sCD40L levels were significantly higher in the PDAC group (30044.2 ± 9747.9 ng/mL) than in the CP (17648.9 ± 7264.0 ng/mL) and normal control (9170.5 ± 5449.8 ng/mL) groups in the initial training dataset (one-way ANOVA, *p* < 0.001; Table 1). Serum sCD40L levels were also higher in the CP group than in the normal group (post-hoc Bonferroni*, p* < 0.001). Similarly, serum CA19-9 levels tended to increase along the PDAC carcinogenic process (*p* < 0.001; Table 1). However, serum CEA levels were not significantly different among three groups (*p* = 0.428; Table 1).

**Table 1:** Serum levels of sCD40L, CA19-9, and CEA in both training and independent validation datasets

**(Training set)**	**Groups (n)**			
**Values**	**Normal (n = 25)**	**CP**^**†**^**(n = 25)**	**PDAC (n = 25)**	***p-*** **value**^**‡**^
Serum sCD40L (ng/ml)	9170.5 ± 5449.8*	17648.9 ± 7264.0	30044.2 ± 9747.9	< 0.001
Serum CA19-9 (U/ml)	7.9 ± 7.1	136.8 ± 329.8	5795.4 ± 7842.7	< 0.001
Serum CEA (ng/ml)	2.3 ± 1.5	2.9 ± 2.0	367.9 ± 1709.4	0.428
**(Validation set)**	**Groups (n)**			
**Values**	**Normal (n = 30)**	**CP (n = 30)**	**PDAC (n = 55)**	***p-*** **value**^**‡**^
Serum sCD40L (ng/ml)	10540.1 ± 5159.9	18709.4 ± 8786.6	27924.6 ± 10202.9	< 0.001
Serum CA19-9 (U/ml)	8.5 ± 7.5	119.6 ± 299.7	3932.7 ± 6839.2	< 0.001
Serum CEA (ng/ml)	2.2 ± 1.4	2.6 ± 2.0	271.4 ± 1246.8	0.359

sCD40L, soluble CD40 ligand; CA19-9, carbohydrate antigen 19–9; CEA, carcinoembryonic antigen; CP, chronic pancreatitis; PDAC, pancreatic ductal adenocarcinoma.

*All tested values are expressed as the mean ± standard deviation.

^†^We included CP patients as the high-risk group of PDAC.

^‡^One-way ANOVA test with the multiple comparisons using the post-hoc Bonferroni method is applied to compare the differences in the means among three disease groups.

*p* < 0.05 (two-tailed) was considered to be statistically significant.

### Diagnostic accuracy of sCD40L for PDAC prediction in training set

**Figure 1:**
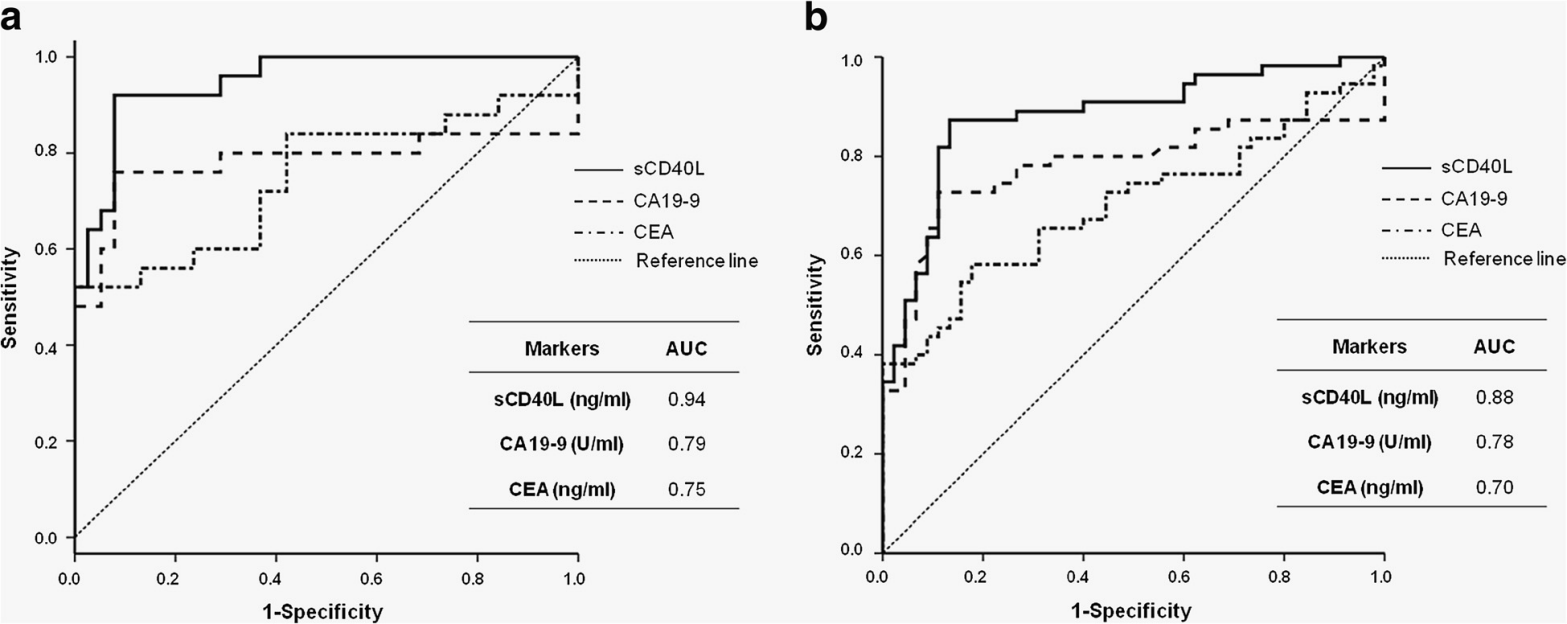
**ROC curves of sCD40L, CA19-9, and CEA for PDAC screening. (a)** Training datasets. **(b)** Validation datasets. AUC, area under the ROC curve.

To compare the diagnostic accuracy of serum sCD40L for PDAC compared with serum CA19-9 and CEA in the training dataset, ROC curves were generated and AUC was calculated (Figure 1a). Serum sCD40L exhibited superior diagnostic potential for PDAC compared with serum CA19-9 and CEA in the training dataset (Figure 1a).

### Serum sCD40L levels along the PDAC carcinogenic sequence in independent validation dataset

Next, we tested whether our results from the training dataset were reproducible by evaluating the diagnostic potential of serum sCD40L for PDAC in an independent validation dataset. The characteristics of patients in validation dataset were shown in supplementary Additional file 1. Consistent with the results of training dataset, serum sCD40L levels in the validation dataset were significantly higher in the PDAC group (27924.6 ± 10202.9 ng/mL) than in the CP (18709.4 ± 8786.6 ng/mL) and normal (10540.1 ± 5159.9 ng/mL) groups (*p* < 0.001; Table 1). And serum sCD40L levels were also higher in the CP group than in the normal group (*p* < 0.001).

### Diagnostic potential of sCD40L for predicting PDAC in the validation dataset

ROC curve also exhibited the superior diagnostic potential of serum sCD40L for predicting PDAC compared with serum CA19-9 or CEA in the validation dataset (Figure 1b). Logistic regression in the validation dataset demonstrated that sCD40L had remarkable diagnostic potential for PDAC screening, either alone or as part of a multiple-marker panel (Table 2). When sCD40L was combined with CA19-9, the specificity increased to 89.1% at 80% sensitivity (cut-off point, 0.35; Table 2). When all of sCD40L, CA19-9, and CEA were used in combination, the sensitivity and specificity in screening for PDAC were remarkably increased (90.9% specificity at 84.4% sensitivity; Table 2).

**Table 2:** Diagnostic accuracy of combinations of serum sCD40L, CA19-9, and/or CEA to detect PDAC by logistic regression in the validation dataset

Marker panel*	**Cut-off point**^**†**^	**Sensitivity**^**‡**^	**Specificity**
sCD40L	0.45	80.0%	85.5%
CA19-9	0.35	80.0%	72.7%
CEA	0.45	68.9%	60.0%
sCD40L + CA19-9	0.35	80.0%	89.1%
sCD40L + CEA	0.60	80.0%	85.5%
CA19-9 + CEA	0.41	80.0%	70.9%
sCD40L + CA19-9 + CEA	0.45	84.4%	90.9%

sCD40L, soluble CD40 ligand; CA19-9, carbohydrate antigen 19–9; CEA, carcinoembryonic antigen; PDAC, pancreatic ductal adenocarcinoma.

*Each marker is included as a linear term and evaluated as a panel from one to three markers combination.

^†^Cut-off point refers to the probability cut-off point to classify subjects as having PDAC or non-PDAC in binary logistic regression.

^‡^For comparison among panels, the cut-off point ensures a target sensitivity of around 80%.

### Correlations between serum sCD40L and clinicopathological characteristics of PDAC

We analyzed the relationships between serum sCD40L and clinicopathological characteristics of PDAC, compared with CA19-9, in the validation dataset (Table 3). Clinically, serum sCD40L was not affected by gender (Spearman’s correlation; γ_s_ = -0.046, *p =* 0.740) or age (γ_s_ = 0.076, *p* = 0.581). Pathologically, serum sCD40L was not significantly correlated with T-stage (γ_s_ = 0.194, *p =* 0.156), lymph node metastasis (γ_s_ = -0.146, *p* = 0.289), or tumor size (γ_s_ = 0.035, *p* = 0.801). However, sCD40L was significantly correlated with distant metastasis (γ_s_ = 0.294, *p =* 0.030), overall stage (γ_s_ = 0.319, *p =* 0.017), and unresectability (γ_s_ = 0.342, *p =* 0.011) while CA19-9 was correlated with nodal metastasis (γ_s_ = 0.362, *p =* 0.008), distant metastasis (γ_s_ = 0.425, *p =* 0.002), and overall stage (γ_s_ = 0.431, *p =* 0.001).

**Table 3:** Relationships between serum sCD40L and clinicopathological characteristics of PDAC, compared with CA19-9 in the validation dataset

	**sCD40L**	**CA19-9**
	**γ**_**s **_**(*p-*** **value)**	**γ**_**s **_**(*p-*** **value)**
Gender (Male: Female)	-0.046 (0.740)	-0.213 (0.126)
Age (≤40, > 40 & ≤60, > 60)	0.076 (0.581)	-0.181 (0.194)
T-stage (T1, T2, T3 ,T4)	0.194 (0.156)	0.095 (0.501)
Node metastasis (N0, N1)	-0.146 (0.289)	**0.362 (0.008)**
Distant metastasis (M0, M1)	**0.294(0.030)**	**0.425 (0.002)**
Overall stage (I, II, III, IV)	**0.319 (0.017)**	**0.431 (0.001)**
Size (≤2 cm, > 2 cm & ≤5 cm, > 5 cm)	0.035 (0.801)	0.179 (0.201)
Unresectability*	**0.342 (0.011)**	0.269 (0.051)

sCD40L, soluble CD40 ligand; CA19-9, carbohydrate antigen 19–9; PDAC, pancreatic ductal adenocarcinoma.

γ_s_, Spearman’s correlation coefficient.

*p* < 0.05 (two-tailed) was considered to be statistically significant.

Statistically significant values are given in bold type.

*A tumor is defined as unresectable when a tumor invades celiac axis or superior mesenteric artery (T4, N0-1, M0, stage III), or metastasizes to distant sites (T1-4, N0-1, M1, stage IV).

### Correlations between serum sCD40L and pro-angiogenic or immunosuppressive cytokines in patients with PDAC in the validation dataset

The results that serum sCD40L levels were correlated with unresectability and distant metastasis of PDAC indicate that sCD40L may be involved in PDAC progression as well as PDAC development. Previous report showing the role of sCD40L in inducing the angiogenesis-associated cytokines [14] also supports our hypothesis. Based on the previous and our current results, we hypothesized that sCD40L may affect the production of pro-angiogenic cytokines during PDAC carcinogenesis, and thereby contribute to promote the distant metastasis of PDAC as well as PDAC development. As expect, serum sCD40L was closely correlated with serum pro-angiogenic cytokines, EGF (Pearson’s correlations; γ_p_ = 0.590, *p <* 0.001), VEGF (γ_p_ = 0.389, *p <* 0.001), and IL-8 (γ_p_ = 0.195, *p =* 0.037; Table 4) when the entire subjects of validation dataset were evaluated. Serum sCD40L was also correlated with serum EGF (γ_p_ = 0.397, *p =* 0.003) and VEGF (γ_p_ = 0.305, *p =* 0.026), and it tended to be correlated with serum IL-8 (γ_p_ = 0.257, *p =* 0.058) when only PDAC patients were evaluated.

**Table 4:** Relationships between serum sCD40L and pro-angiogenic or immunosuppressive cytokines in patients with PDAC in the validation dataset

**(All groups)**		**EGF**	**VEGF**	**IL-8**	**IL-6**	**IL-10**	**IL-1RA**
**Serum sCD40L**	**(γ**_**p**_**)**	0.590	0.389	0.195	0.203	0.215	0.243
	(*p-*value)	**< 0.001**	**< 0.001**	**0.037**	**0.029**	**0.021**	**0.009**
**(PDAC group)**		**EGF**	**VEGF**	**IL-8**	**IL-6**	**IL-10**	**IL-1RA**
**Serum sCD40L**	**(γ**_**p**_**)**	0.397	0.305	0.257	0.209	0.224	0.248
	(*p-*value)	**0.003**	**0.026**	0.058	0.126	0.100	0.068

Pro-angiogenic cytokines, EGF, VEGF, and IL-8; immunosuppressive cytokines, IL-6, IL-10, and IL-1RA.

γ_p_, Pearson’s correlation coefficient.

*p* < 0.05 (two-tailed) was considered to be statistically significant.

Statistically significant values are given in bold type.

Previous studies also suggested that elevated serum sCD40L levels play an immunosuppressive role in cancer patients [20]. Thus, we also evaluated correlations between serum sCD40L and serum anti-inflammatory immunosuppressive cytokines (IL-6, IL-10, and IL-1RA). When all cases were evaluated, serum sCD40L was positively correlated with serum IL-6 (γ_p_ = 0.203, *p =* 0.029), IL-10 (γ_p_ = 0.215, *p =* 0.021), and IL-1RA (γ_p_ = 0.243, *p =* 0.009; Table 4). For only PDAC patients, serum sCD40L was not significantly correlated with IL-6 (γ_p_ = 0.209, *p =* 0.126), IL-10 (γ_p_ = 0.224, *p =* 0.100), or IL-1RA (γ_p_ = 0.248, *p =* 0.068). However, there was a tendency toward positive correlations with serum sCD40L levels. These results suggest that sCD40L affects PDAC development through induction of both immunosuppressive cytokines (IL-6, IL-10, and IL-1RA) and pro-angiogenic cytokines (EGF, VEGF, and IL-8), in agreement with previous studies [20,26]. And, sCD40L also may influence on PDAC metastasis, mainly through induction of pro-angiogenic cytokines (EGF, VEGF, and IL-8), and partially immunosuppressive cytokines (IL-6, IL-10, and IL-1RA).

### Diagnostic advantage of serum sCD40L to overcome the limitations of CA19-9

Serum CA19-9 is the most widely used biomarker for PDAC, but it sometimes does not correspond to a disease entity, making it difficult to differentiate between cancer and non-cancer conditions. Thus, we evaluated whether serum sCD40L could differentiate PDAC from benign conditions in cases where the levels of CA19-9 are inappropriately high in normal subjects or inappropriately low in patients with PDAC in the validation dataset. First, we compared the serum levels of sCD40L and CA19-9 between the cancer and non-cancer groups in subjects with low-serum CA19-9 levels (≤40 ng/mL) and those with high-serum CA19-9 levels (>40 ng/mL). Serum sCD40L levels were definitively different between the cancer and non-cancer groups in both low-CA19-9 and high-CA19-9 groups independent of serum CA19-9 levels (each *p* < 0.001; data not shown); this means that serum sCD40L can differentiate between PDAC and non-cancer in cases where serum CA19-9 levels were not significantly different between cancer and non-cancer groups. Logistic regression showed that serum sCD40L exhibited remarkable diagnostic potential to differentiate between PDAC and non-cancer conditions in both the low and high CA19-9 level groups (Table 5).

**Table 5:** Diagnostic advantages of serum sCD40L for differentiation of PDAC from non-cancer in cases where the levels of CA19-9 are inappropriate^*^

	**Low-serum CA 19–9 subjects**			**High-serum CA 19–9 subjects**		
	**Cut-off value**	**Sensitivity**	**Specificity**	**Cut-off value**	**Sensitivity**	**Specificity**
**sCD40L (ng/ml)**	20000	80.0%	86.7%	20000	77.0%	85.0%

Sensitivity and specificity of serum sCD40L to differentiate PDAC from non-cancer in subjects with low-serum (≤40 ng/mL) or high-serum CA19-9 (>40 ng/mL) levels when serum CA19-9 does not correspond to a disease entity.

sCD40L, soluble CD40 ligand; CA19-9, carbohydrate antigen 19–9; CEA, carcinoembryonic antigen; PDAC, pancreatic ductal adenocarcinoma.

^*^ This means that the cases when CA19-9 are inappropriately high in normal subjects or inappropriately low in patients with PDAC in the validation dataset.

### Prediction of PDAC prognosis by serum sCD40L levels

**Figure 2:**
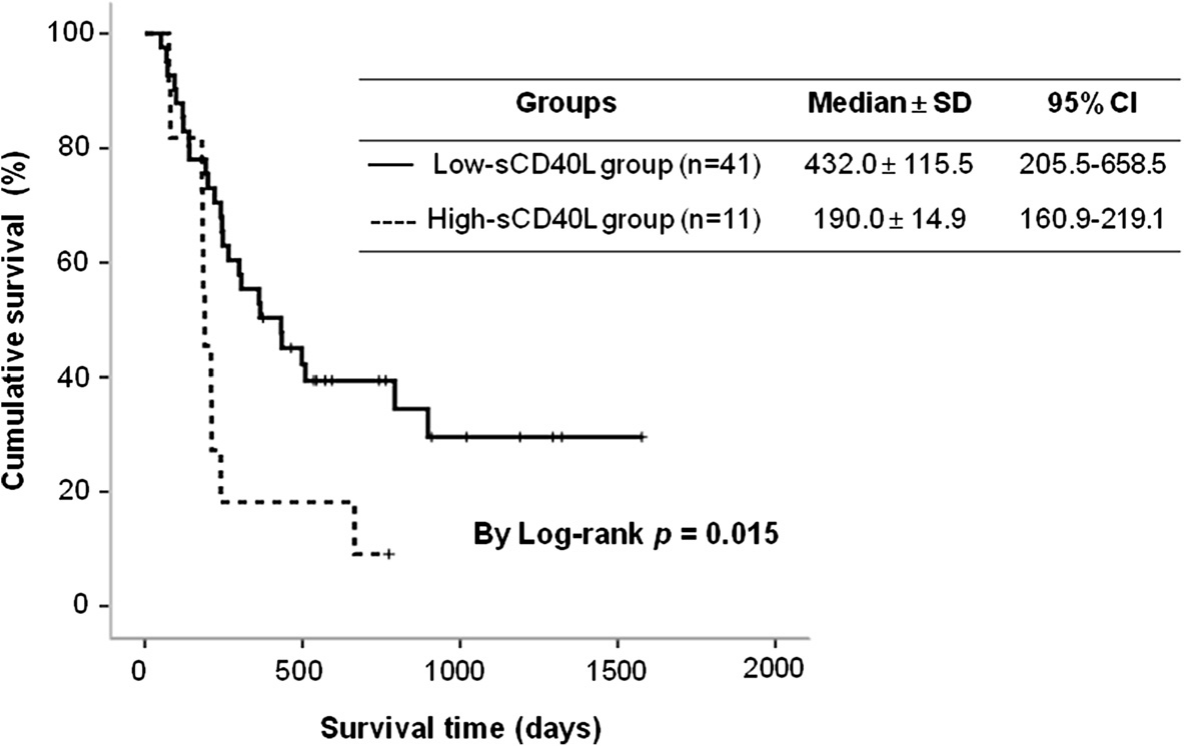
**Kaplan-Meier survival curves for PDAC patients with high-serum (>35,000 ng/ml) vs. low-serum (≤35,000 ng/mL) sCD40L levels.** Log-rank test, *p* = 0.015. SD, standard deviation; CI, confidence interval.

Fifty-two PDAC patients in the validation dataset were included in the final survival analysis. Three of the original 55 patients were excluded due to follow-up loss. The maximum follow-up time was 1,576 days, and the median follow-up time was 282 days. Kaplan-Meier survival curves showed that PDAC patients with high-levels of serum sCD40L (>35,000 ng/mL) had a significantly worse prognosis than those with low-levels (≤35,000 ng/mL, log-rank, *p* = 0.015; Figure 2). The median survival was 190 days (95% confidence interval [CI], 160.9–219.1 days) in patients with high-levels of serum sCD40L versus 432 days (95% CI, 205.5-658.5) in patients with low-levels.

Univariate Cox proportional-hazards regression model showed that distant metastasis, unresectability, large tumor size, low Karnofsky performance status scale, high-serum CEA (>3 ng/mL) levels, and high-serum sCD40L (>35,000 ng/mL) levels were statistically significant poor prognostic factors for PDAC (Table 6). The hazard ratio (HR) of high-serum sCD40L levels for mortality was 2.468 (95% CI, 1.162–5.242; *p* = 0.019).

**Table 6:** Univariate and adjusted-multivariate Cox proportional-hazards estimates for patients with PDAC in the validation dataset

**Univariate analysis**	**HRs**	**95% CI**	***p*-value**
Gender (male vs. female)	0.583	0.280-1.211	0.148
Age (≤40, > 40 & ≤60, > 60)	0.649	0.359-1.175	0.154
T-stage (T1, T2, T3 ,T4)	1.428	0.902-2.261	0.129
Node metastasis (N0, N1)	1.960	0.810-4.745	0.136
Distant metastasis (M0, M1)	3.558	1.695-7.469	0.001
Unresectability (resectable vs. unresectable)	2.917	1.025-8.298	0.045
Tumor Size (≤2 cm, > 2 cm & ≤ 5 cm, > 5 cm)	2.419	1.191-4.917	0.015
Histological differentiation (Well, Moderate, Poor; n)	1.154	0.777-1.714	0.478
Karnofsky performance status scale (continuous variable)*	0.962	0.936-0.988	0.005
High-serum CA19-9 (≤40 U/ml vs. > 40 U/ml)	1.811	0.788-4.160	0.162
High-serum CEA (≤3 ng/ml vs. > 3 ng/ml)	2.137	1.062-4.298	0.033
High-serum sCD40L (≤35,000 ng/ml vs. > 35,000 ng/ml)	2.468	1.162-5.242	0.019
**Multivariate analysis**	**HRs**^**†**^	**95% CI**	***p*-value**
Distant metastasis (M0, M1)	3.469	1.375-8.749	0.008
Unresectability (resectable vs. unresectable)	0.588	0.168-2.060	0.406
Tumor Size (≤2 cm, > 2 cm & ≤ 5 cm, > 5 cm)	2.454	1.075-5.602	0.033
Karnofsky performance status scale (continuous variable)*	0.969	0.940-0.998	0.038
High-serum CEA (≤3 ng/ml vs. > 3 ng/ml)	1.354	0.549-3.340	0.511
High-serum sCD40L (≤35,000 ng/ml vs. > 35,000 ng/ml)	2.509	1.038-6.067	0.041

HR, hazard ratio of mortality; CI, confidence interval; sCD40L, soluble CD40 ligand; CA19-9, cancer antigen 19–9; CEA, carcinoembryonic antigen.

*p*-value of < 0.05 is considered to be statistically significant.

*This scale was evaluated at diagnosis before treatment start.

^†^Hazard ratios (HRs) were adjusted for other variables by the “enter” method in multivariate Cox analysis.

Multivariate Cox proportional-hazards regression model demonstrated that distant metastasis, large tumor Size, low Karnofsky performance status scale, and high-serum sCD40L remained as significant poor prognostic factors from the univariate analysis after adjustment (Table 6). The adjusted HR for high-serum sCD40L levels was 2.509 (95% CI, 1.038-6.067; *p* = 0.041).

## Discussion

This study evaluated the role of sCD40L in PDAC patients using prospectively collected human serum samples. To our knowledge, this study is the first report to validate serum sCD40L as a potential diagnostic and prognostic biomarker for PDAC. We also provide the possible clinical evidence that elevated serum sCD40L levels is linked to PDAC development and metastasis through neoangiogenesis and immunosuppression. We validated the predictive potentials of serum sCD40L for PDAC in a training dataset and confirmed the reproducibility in an independent dataset. We also followed the Standards for Reporting of Diagnostic Accuracy (STARD) statement [27] and the Reporting recommendations for tumor marker prognostic studies (REMARK) guideline [28].

Serum sCD40L levels were significantly elevated in PDAC patients compared with age- and gender-matched non-cancer subjects in training and validation datasets, which result indicates serum sCD40L as a promising biomarker candidate for PDAC. ROC curve and estimated sensitivity and specificity by logistic regression support our hypothesis (Figure 1 and Table 2). Serum sCD40L showed stronger PDAC prediction power than CA19-9 and CEA. Nevertheless, the use of serum sCD40L as a single biomarker for PDAC may be limited because serum sCD40L can be elevated in other cancers [20-22] and certain benign disorders including cardiovascular diseases or immuno-inflammatory diseases [9,10]. Therefore, combining sCD40L with pre-existing biomarkers for PDAC, such as CA19-9 and/or CEA would overcome this limitation by increasing specificity for PDAC. As expected, combination of serum sCD40L with CA19-9 increased the specificity nearly 90% at a sensitivity of 80%. Combining all of sCD40L, CA19-9, and CEA produced the highest sensitivity and specificity (90.9% specificity at 84.4% sensitivity; Table 2), confirming that serum sCD40L is a valuable diagnostic biomarker as both alone and part of multiple-marker panels. Additionally, serum sCD40L was able to overcome the limitations of CA19-9 in patients where CA19-9 did not correspond to a disease entity, such as in the cases of benign biliary obstruction or combined benign liver diseases. Serum sCD40L also aids differentiation between resectable and unresectable cases, and negative and positive distant metastasis. Therefore, serum sCD40L can provide insight into whether PDAC is curable and help avoid unnecessary surgical procedures.

Kaplan-Meier survival analysis and Cox proportional-hazards regression models indicated that PDAC patients with high-serum sCD40L (>35,000 ng/mL) levels had poorer prognosis than those with low-serum sCD40L (≤35,000 ng/mL) levels in agreement with previous reports [29].

Many past studies have tried to explain the pathogenesis of sCD40L-related carcinogenesis and progression. Although early studies addressed the pro-apoptotic capacity of the CD40-CD40L pair [30], subsequent studies suggested that sCD40L may induce an anti-apoptotic signal under certain stimuli [31]. Several studies have also suggested that cancer-mediated thrombin generation by interaction between tumor cell CD40 and platelet-derived sCD40L is involved in tumor cell growth, motility, and angiogenesis through activation of gene transcription that promotes tumor metastasis and angiogenesis [32,33]. In tumor-prone transgenic mice, CD40-mediated neovascularization was essential for early-stage tumorigenesis [34]. Recently, serum sCD40L was found to negatively regulate cancer immune responses [20].

Like previous studies, we observed that serum sCD40L levels correlated with several well-known pro-angiogenic cytokines including EGF, VEGF, and IL-8 when either all subjects or only PDAC patients were evaluated. On the other hand, immunosuppressive cytokines including IL-6, IL-10, and IL-1RA exhibited strong positive correlations with sCD40L when all subjects were evaluated, although there were weak positive correlations when only PDAC patients were analyzed. These results imply that sCD40L may induce the pro-angiogenic cytokines production, which can affect both PDAC development and metastasis like previous studies [32-34]. Also, circulating sCD40L may enhance immunosuppressive cytokines production, which can affect mainly PDAC development disturbing tumor immune surveillance. To confirm the causal relationship between serum sCD40L and these cytokines during PDAC development and progression, well-designed cell biological analysis and animal experiments are further needed.

Although the sample sizes achieved > 80% statistical power in all tested datasets, and we also validated our findings of initial training dataset in independent validation datasets, the sample sizes of present study is relatively small because of the difficulty of pathological confirmation of PDAC and resultant limitation of patient enrollment. Therefore, further large-scale studies should follow to confirm our current results.

## Conclusion

We demonstrated the clinical significance of sCD40L in PDAC development and progression using prospectively collected human serum samples, and we validated serum sCD40L as a useful serum biomarker for PDAC by demonstrating its predictive and prognostic potentials and its ability to overcome the limitations of serum CA19-9. We hope that current study can help the early diagnosis of PDAC and improvement of prognosis of PDAC.

## Competing interests

The authors declare that they have no competing interests.

## Authors’ contributions

HWC and JBL contributed the conception and design of the study. HWC and JBL collected and stored all the samples. JBL acquired the quantitative data of serum sCD40L and cytokines concentration. HWC conducted statistical analysis of all data. JBL supervised all experiments. HWC and JBL drafted the manuscript. Both authors have given final approval of the version to be published and have agreed to be accountable for all aspects of the work.

## Supplementary Material

Additional file 1 Clinicopathological features of subjects in the validation dataset. Click here for file
