# Supplementary material for: Clinical significance of elevated serum soluble CD40 ligand levels as a diagnostic and prognostic tumor marker for pancreatic ductal adenocarcinoma
Source: J Transl Med. 2014 Apr 21;12:102. doi: 10.1186/1479-5876-12-102 (PMC4021610; doi:10.1186/1479-5876-12-102)
Supplement: Additional file 1 — Clinicopathological features of subjects in the validation dataset. [file 1479-5876-12-102-S1.doc]

**Additional file 1 Clinicopathological features of subjects in the validation dataset.**

|  | Disease groups (n) | | |  |
| --- | --- | --- | --- | --- |
| Clinicopathological features | Normal (30) | CP (30) | PDAC (55) | |
| Gender (male vs. female; n) | 20:10 | 18:12 | 36:19 | |
| Age (mean ± SD; years) | 56.3±12.3 | 53.3±11.4 | 59.6±10.8 | |
| T-stage (T1 : T2 : T3 : T4; n) | NS | NS | 1:5:17:32 | |
| Node metastasis (N0 vs. N1; n) | NS | NS | 13:42 | |
| Distant metastasis (M0 vs. M1; n) | NS | NS | 26:29 | |
| Overall stage (I : II : III : IV; n) | NS | NS | 4:8:14:29 | |
| Size (≤ 2cm : > 2cm & ≤ 5cm : > 5cm; n) | NS | NS | 1:15:39 | |
| Resectable:Unresectable (n)*^*^* | NS | NS | 12:43 | |
| Histological differentiation (well : moderate : poor; n) | NS | NS | 14:13:28 | |
| Karnofsky performance status scale^†^ | NS | NS | 75.2±13.2 | |

CP, chronic pancreatitis; PDAC, pancreatic ductal adenocarcinoma; SD, standard deviation; NS, not studied;

^*^A tumor is defined as unresectable when a tumor invades celiac axis or superior mesenteric artery (T4, N0-1, M0, stage III), or metastasizes to distant sites (T1-4, N0-1, M1, stage IV). .

^†^This scale was evaluated at diagnosis before treatment start.
